# Peritoneovenous Shunting to Manage Chylous Ascites Following Liver Resection for Cholangiocarcinoma: A Case Report

**DOI:** 10.70352/scrj.cr.25-0616

**Published:** 2025-11-12

**Authors:** Mizuki Nakajima, Kuniya Tanaka, Sae Morioka, Akihiro Nakamura, Kenichi Matsuo, Yuki Takahashi

**Affiliations:** Department of General and Gastroenterological Surgery, Showa Medical University Fujigaoka Hospital, Yokohama, Kanagawa, Japan

**Keywords:** chylous ascites, liver resection, peritoneovenous shunt

## Abstract

**INTRODUCTION:**

Liver resection has rarely been reported as a cause of chylous ascites. Such ascites, consisting mainly of hepatic lymph, is usually caused by injury to the lymphatic system between the hepatic hilum and the hepatoduodenal ligament. We report a patient who developed chylous ascites after liver resection and required a peritoneovenous shunt.

**CASE PRESENTATION:**

An 80-year-old man with a liver tumor diagnosed as cholangiocarcinoma underwent an extended right hemihepatectomy with lymphadenectomy following right portal vein embolization. Postoperatively, he developed chylous ascites that resolved with dietary measures, bowel rest, and administration of octreotide. However, he was readmitted a month after discharge with abdominal distension and dyspnea from massive ascites and pleural effusion. Abdominal paracentesis confirmed chylous ascites, showing a triglyceride concentration of 614 mg/dL. After failure of conservative therapy, including dietary and pharmacologic interventions, peritoneovenous shunting was performed. Clinical status improved after shunting, with no adverse events except for transient fever.

**CONCLUSIONS:**

Refractory chylous ascites after liver resection should be treated promptly with surgical measures such as peritoneovenous shunting to maintain the patient’s general condition.

## Abbreviations

ALBI
: albumin–bilirubin score
ALPPS
: associating liver partition and portal vein ligation for stage hepatectomy
FLR
: future liver remnant
GSA
: 99mTc-galactosyl serum albumin
ICG
: indocyanine green
ICGR15
: indocyanine green retention rate at 15 min
KICG
: rate of ICG disappearance from plasma
LHL15
: uptake ratio of the liver to that of the liver and heart at 15 min
PV
: portal vein
remKICG
: KICG for the future liver remnant
rt.
: right
S
: segment
TIPS
: transjugular intrahepatic portosystemic shunt

## INTRODUCTION

Chylous ascites, a rare form of ascites resulting from leakage of lipid-rich lymph into the peritoneal cavity, has been reported to occur in approximately 1 out of 11000 to 20000 hospital admissions.^1,2)^ Such leakage can infrequently complicate abdominal surgery following a wide range of procedures.^3)^ Lymphatic leakage during surgery mainly complicates lymph node dissection and usually resolves spontaneously, representing a favorable overall prognosis.^4,5)^ However, the prognosis of chylous ascites mainly depends on its immediate cause and underlying etiology.^6)^ Massive ascites is frequent and may cause severe malnutrition requiring its own treatment.

Liver resection has rarely been reported as a cause of chylous ascites. However, a huge liver mass can increase lymphatic blockade sufficiently to increase hydrostatic pressure, leading to persistent postoperative chylous leakage.^7)^ Portal hypertension from underlying liver disease, such as cirrhosis, can also elevate lymphatic pressure sufficiently to cause endothelial compromise or rupture of dilated serosal lymphatic channels, similarly leading to chylous ascites.^8,9)^ Lymph flow is 10 times higher in patients with cirrhosis than in healthy individuals.^10)^ Furthermore, during hepatectomy combined with lymphadenectomy, inadvertent injury to lymphatics in the lowest fold of the hepatoduodenal ligament may lead to persistent postoperative chylous leakage.

Here, we report a case of chylous ascites that developed after an extended right hemihepatectomy following right PV embolization, eventually requiring a peritoneovenous shunt (Denver shunt).

## CASE PRESENTATION

**Fig. 1:**
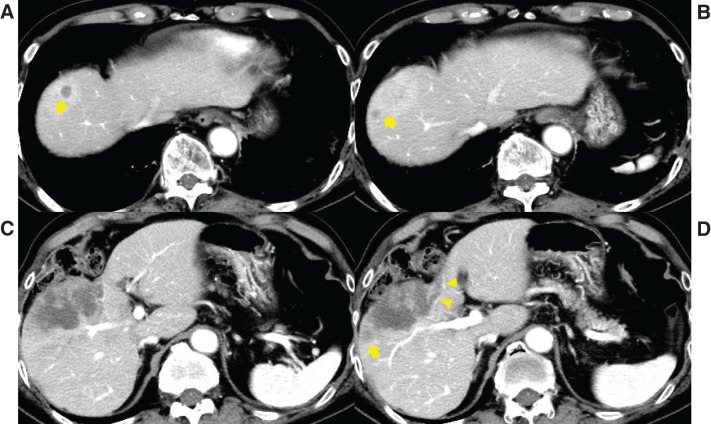
Preoperative CT images of the liver detected a tumor 80 mm in diameter located mainly in the right anterior section (**C**). Small nodules surrounded the main tumor (**A**, **B**, **D**). Extension of thrombus in segment 4 of the portal vein was apparent (**D**). No obvious lymphadenopathy was observed. Arrows indicate likely small intrahepatic metastases. Arrowheads indicate tumor thrombus in the portal vein.

An 80-year-old man with liver tumors diagnosed as cholangiocarcinoma was referred to our institution. The main tumor, measuring 80 mm in diameter, was located mostly in the right anterior section. Surrounding the main tumor, small nodules suspected to be intrahepatic metastases were present. Extension of thrombus within S4 of the PV was also suspected. No obvious lymphadenopathy was noted (**Fig. 1**). Aggressive chemotherapy was contraindicated according to oncologists because of the patient’s age. We decided to perform an extended right hemihepatectomy with lymphadenectomy.

Preoperative hepatic reserve test results included an ICGR15 of 13.87%, while the KICG was 0.132. A GSA-rectified ICG value, determined using a GSA scintigraphic receptor index with the formula 105 − 101 × LHL15,^11)^ was 17.13 (**Table 1**). Serum albumin (3.9 g/dL), total bilirubin (0.4 mg/dL), prothrombin International Normalized Ratio (1.02), platelet count (22.6 × 10^4^/mL), and other routine laboratory values were essentially normal. Liver function was graded as Child–Pugh A, 5 points; the modified ALBI was grade 1.

**Table 1:** Serial changes of the hepatic reserve and the liver volume

	Before PV embolization	Before hepatectomy
Liver volume		
Estimated total liver volume	970 cm^3^	1133 cm^3^
Planned resection volume	580 cm^3^	585 cm^3^
	59.8%	51.6%
FLR volume	390 cm^3^	548 cm^3^
	40.2%	48.4%
Hepatic reserve		
ICGR15	13.87%	11.40%
KICG	0.132	0.144
GSA ICG	17.13	−
ALBI score	−2.76	−2.59
Prognostic evaluation		
Prognostic score	66.5	56.2
remKICG	0.0529	0.0700

Data before hepatectomy were calculated at 21 days after the PV embolization.

ALBI, albumin–bilirubin score; FLR, future liver remnant; GSA, 99mTc-galactosyl serum albumin; ICG, indocyanine green; ICGR15, indocyanine green retention rate at 15 min; KICG, rate of ICG disappearance from plasma; PV, portal vein; remKICG, KICG for the future liver remnant

Removal of the tumor required right hepatectomy extending to the left medial section. Estimated resection volume, resection volume relative to total liver volume, and planned remnant liver volume were 580 cm^3^, 59.8%, and 390 cm^3^, respectively (**Table 1**). Prediction scores^12)^ for the planned procedure, calculated using the formula −84.6 + 0.933a + 1.11b + 0.999c, with a representing anticipated resection fraction (%); b, ICGR15 (%); and c, patient age in years, were 66.5 using ICG R15 and 70.1 using GSA-rectified ICG. Further, remKICG, calculated using the formula, KICG × % volume of FLR /100,^13)^ was 0.0529 (**Table 1**). The patient’s advanced age, a prediction score over 60, and a remKICG of nearly 0.05 indicated that any adequate procedure performed as a single stage would not be tolerated considering FLR function. Therefore, we chose to perform right PV embolization.

Guided by imaging, right PV embolization was performed under general anesthesia via an ileocolic vein approach. Embolic materials were introduced using a 6-Fr balloon catheter guided by ongoing imaging. The materials and doses used were 1 g of gelatin pellets (Gelfoam powder; Upjohn, Kalamazoo, MI, USA), 10–20 mL of oleic acid monoethanolamide (Oldamine; Grelan, Tokyo, Japan), and 20 mL of diatrizoate sodium meglumine (76% Urografin; Schering, Berlin, Germany).

**Fig. 2:**
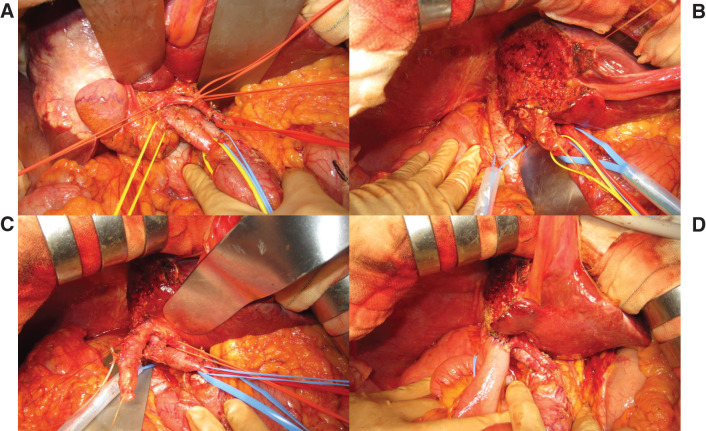
Intraoperative findings. Lymphadenectomy preserving the extrahepatic bile duct was performed (**A**). Appearance after removal of the tumor-involved area and surrounding tissue on the right side of the liver (**B**). Findings during (**C**) and after (**D**) resection of the extrahepatic duct and reconstruction by hepatojejunal anastomosis.

**Fig. 3:**
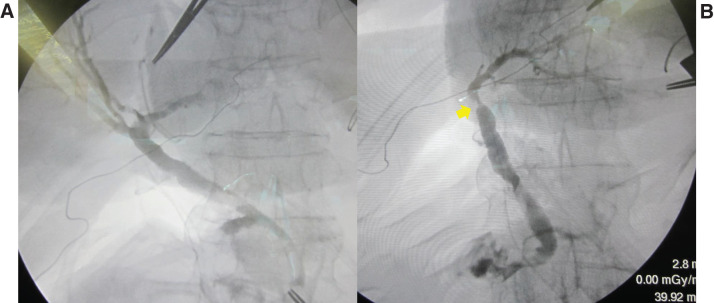
Intraoperative cholangiographic findings. Appearance before (**A**) and after (**B**) division of the right bile duct. Stricture of the hilar bile duct was confirmed (arrow in **B**).

Dynamic CT was performed 1 and 3 weeks after PV embolization to assess remnant liver hypertrophy and PV thrombus extension. After 3 weeks of PV embolization, the estimated resection volume relative to total liver volume had decreased to 51.6% (585 cm^3^). FLR volume had increased to 548 cm^3^, and remKICG had increased to 0.0700. The prediction score had decreased to 56.2. Remnant liver hypertrophy was considered likely to become sufficient. Accordingly, right hemihepatectomy extended to S4 and accompanied by lymphadenectomy was performed 4 weeks after PV embolization. Initially, lymphadenectomy was performed while preserving the extrahepatic bile duct (**Fig. 2A** and **2B**), but stricture of the hilar bile duct was confirmed by intraoperative cholangiography (**Fig. 3**) after dividing the right hepatic duct near the confluence of the right and left ducts. We therefore resected the extrahepatic duct and reconstructed it by hepaticojejunal anastomosis (**Fig. 2C** and **2D**). Dissected lymph nodes were located in the hepatoduodenal ligament, along the common hepatic artery, and on the posterior surface of the pancreatic head. A tube for enteral nutrition was inserted intraoperatively to provide early nutritional support. Operative time was 600 min, and intraoperative blood loss was 70 mL. The total clamping time for the hepatoduodenal ligament during parenchymal transection was 58 min. The maximum tumor size in the resected specimen was 80 mm in diameter. Five intrahepatic metastases with diameters of about 10 mm were present near the main tumor. The histopathologic diagnosis was intrahepatic cholangiocarcinoma of the small duct type. The diagnosis according to the general rules for the clinical and pathological study of primary liver cancer^14)^ was: fc(−), fc-inf(−), sf(−), s0, vp0, vv0, va0, b0. The TNM (tumor, lymph node, metastasis) stage was T3N0M0, Stage III. No lymph node metastases were evident, and the margin was free of tumor.

An elemental diet was started on POD 1, and oral intake was initiated on POD 4. The patient’s early postoperative course was uneventful except for a high output of ascitic fluid through the abdominal drain. Ascites was controlled by administration of diuretics, but its content changed from serous to chylous on POD 19, when its triglyceride concentration was 291 mg/dL. Chylous ascites was treated with dietary measures and bowel rest using total parenteral nutrition, along with the administration of octreotide. Ascites was well controlled by these conservative measures, and the patient was discharged from the hospital on POD 46.

**Fig. 4:**
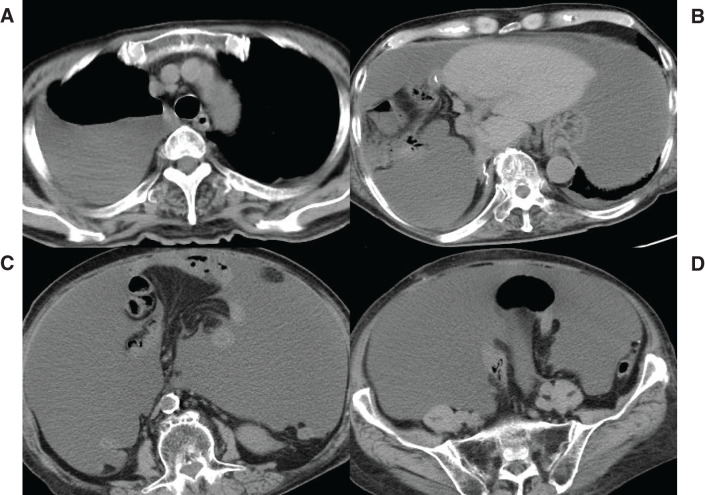
CT findings at readmission. Massive pleural effusion was present, mainly in the right hemithorax (**A**). Ascites surrounded the remnant liver (**B**) and much of the abdomen (**C**), extending to the pelvis (**D**).

**Fig. 5:**
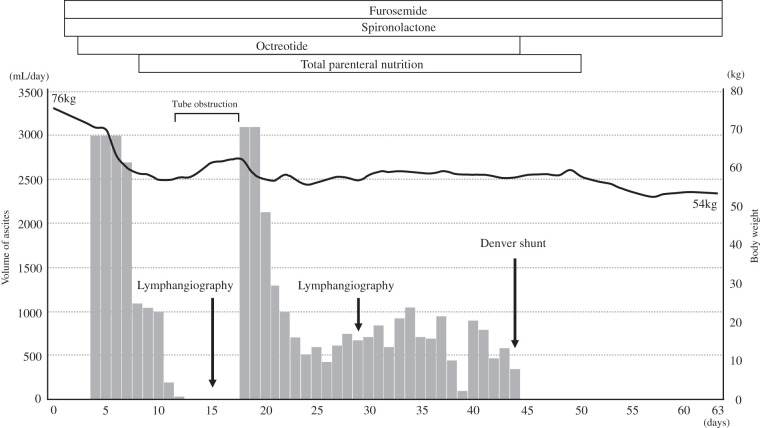
Clinical course after readmission. A reduction in fluid volume within the abdominal paracentesis tube 10–15 days after readmission reflected tube obstruction by viscous chylous ascites. After lymphangiography was performed twice with limited benefit, a successful Denver shunt was placed 44 days after readmission. Daily diuretic doses were 10–20 mg of furosemide, 25 mg of spironolactone, and 300 μg of octoreotide. The duration of diuretic and octreotide administration and total parenteral nutrition is indicated.

**Fig. 6:**
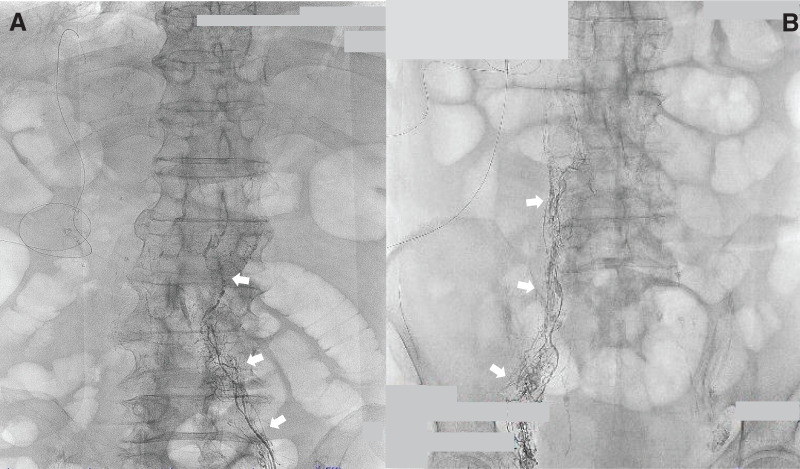
Findings of bilateral lymphangiography. No leakage site was detected by lymphangiography performed via a left inguinal lymph node on day 15 (**A**) and via a right inguinal lymph node on day 29 (**B**).

**Fig. 7:**
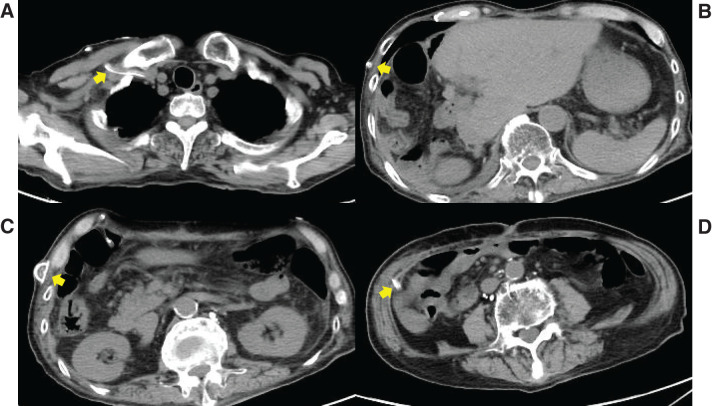
CT findings after placement of a peritoneovenous shunt (Denver shunt). Ascites was diminished after shunting. The shunt tube entered the right subclavian vein (**A**) from the lower abdominal cavity (**D**) via the subcutis (**B**, **C**).

Thirty-four days after hospital discharge, he was readmitted because of dyspnea from abdominal distension with massive ascites, as well as pleural effusion (**Fig. 4**). Laboratory tests showed a slight elevation of C-reactive protein (3.71 mg/dL); other blood test results were normal, including liver function and tumor marker assessments. Abdominal paracentesis results indicated exudation; the Rivalta test was positive, and the triglyceride content of the fluid was 614 mg/dL. Bowel rest by total parenteral nutrition, administration of diuretics, and octreotide administration were promptly initiated after confirmation of chylous ascites. Daily dosages were 10–20 mg of furosemide, 25 mg of spironolactone, and 300 μg of octoreotide. **Figure 5** shows the treatment duration for each drug and serial changes in the patient’s body weight. Dosages of diuretics were adjusted based on serum sodium and potassium concentrations, with sodium maintained at 125–140 mEq/L and potassium at 3.5–5.5 mEq/L. A 20% albumin preparation was given as needed to maintain serum albumin levels above 2.0 g/dL. During treatment, lymphangiography was performed twice via the inguinal lymph nodes, but no site of lymphatic leakage was detected (**Fig. 6**). The volume of chylous ascites did not decrease following lymphangiography. Accordingly, a peritoneovenous shunt (Denver shunt) was created 44 days after readmission. Before shunting, cytology of ascitic fluid was performed twice, confirming that the Papanicolaou class was II with no malignancy. Negative bacterial cultures of ascitic fluid were obtained repeatedly before creation of the shunt. The volume of chylous ascites discharged via the abdominal drain promptly and markedly decreased (**Fig. 7**), permitting oral intake. After his weight had gradually decreased, he was discharged from the hospital 63 days after readmission (**Fig. 5**). Ten days after discharge, a transient fever was noted. This subsided with conservative management, and no signs of infection were apparent. Unfortunately, liver metastasis was evident 10 months after surgery. The patient declined further active treatment, including chemotherapy.

## DISCUSSION

The lymphatic system within the peritoneal cavity can be divided into 3 parts: lumbar, intestinal, and hepatic.^15)^ These regions are the main contributors to the cisterna chyli, a dilated sac within the retroperitoneum at the L1–2 vertebral level. This sac continues cranially as the thoracic duct.^16)^ The hepatic lymphatic system includes an ascending pathway along the hepatic veins and a descending pathway along the hepatoduodenal ligament.^17)^ Chylous ascites typically is caused by rupture of these lymphatics or increased peritoneal lymphatic pressure resulting from obstruction. Underlying etiologies may be traumatic, congenital, infectious, neoplastic, postoperative, cirrhotic, or cardiogenic.^18)^ Ascites consisting mainly of hepatic lymph usually results from injury to the lymphatic system from the hepatic hilum to the hepatoduodenal ligament.^19)^

For diagnosis, CT is the best imaging modality for evaluating intraperitoneal fluid accumulations, but unfortunately it cannot differentiate between chylous and clear ascites given their identical densities. Many studies have established that an elevated ascitic fluid triglyceride concentration is the best way to establish a diagnosis of chylous ascites. The current consensus accepts a value exceeding 200 mg/dL as diagnostic.^20,21)^ Lymphangiography can detect abnormal retroperitoneal nodes, fistulas, and leakage from lymphatic channels, as well as identify the site of the leak. Studies have reported a detection rate of 64%–86% for leakage sites in patients with chylothorax and chylous ascites.^22)^ Pedal and intranodal lymphangiography can visualize lumbar and paraaortic lymphatic leakage, but conventional lymphangiography cannot detect leakage sites in hepatic lymphatic vessels. Such hepatic lymphorrhea can be visualized only by transhepatic lymphangiography.^23)^ In our patient, ascitic fluid triglyceride concentrations were 291 mg/dL immediately after the operation and 614 mg/dL at readmission. Leakage from the lymphatic system could not be detected by conventional lymphangiography via the inguinal lymph nodes. Considering the surgical procedure, extended hemihepatectomy with lymphadenectomy of the hepatoduodenal ligament, and no detection of leakage site by conventional lymphangiography, disruption of the patient’s hepatic lymphatic system during lymphadenectomy was considered the cause of his chylous ascites.

Nonsurgical treatments for chylous ascites include dietary measures as well as pharmacologic agents such as somatostatin and octreotide. Although no obvious relationship between the resumption of diet and chylous ascites was evident in our patient, we initially carried out dietary and pharmacologic measures. Cases unresponsive to medical management usually require procedures such as percutaneous embolization or lymphangiography. The latter may have therapeutic as well as diagnostic benefits. The mechanism by which lymphangiography can reduce lymphatic leaks has not been fully elucidated, but extravasation of lipiodol, an ethiodized oil contrast agent, can induce a granulomatous inflammatory reaction that reduces leakage.^18)^ Radiotherapy is another treatment option used to induce localized fibrosis, aiming to seal lymphatic leaks.^24)^ This approach would have been difficult in our patient since the leakage site was unclear. Indications for surgery include chylous leakage exceeding 1000 mL/day for over 5 days or a leak persisting for over 2 weeks despite optimal conservative management. Surgery may also be considered when serious nutritional or metabolic complications develop.^25)^

Even if the lymphatic duct in the hepatoduodenal ligament is damaged during surgery, lymphatic flow usually loops around the anastomotic branch, or an anastomosis of lymphatic channels occurs upon occlusion of the damaged channel.^19)^ However, our patient did not benefit after readmission despite bowel rest with total parenteral nutrition and the addition of octreotide. More than 1000 mL/day of chylous leakage continued for over 5 days, so surgical treatment, including percutaneous embolization and surgical ligation of injured lymphatic vessels, was required. However, the inability to identify a leakage site, as well as poor general condition and hepatic reserve, made treatment difficult. Immediately before creation of the Denver shunt, our patient’s nutritional status was poor, reflected by a serum albumin concentration of 1.8 g/dL and a prognostic nutritional index^26)^ of 24.45. We speculated that our patient’s massive ascites was caused not only by chyle but also by portal hypertension following the extended liver resection. Finally, the unknown leakage site essentially precluded aggressive surgery.

Important treatment modalities not suitable for our patient include a TIPS and peritoneovenous shunts such as a LeVeen or Denver shunt.^18)^ TIPS, which creates a communication between the portal and systemic circulations within the liver, has been shown to relieve lymphatic hypertension by reducing portal pressure.^20,27)^ In cases where portal hypertension dominates the pathophysiology, TIPS would be more appropriate than peritoneovenous shunting. In our patient, however, TIPS would have been hazardous since the liver remnant was largely limited to the left lateral section and the severity of portal hypertension was unknown. A peritoneovenous shunt can be a good option for patients who are refractory to medical therapy and who are poor candidates for major surgery. The shunt returns chylous fluid to the circulating blood, which can improve hemodynamic, nutritional, and immunologic status.^28)^ Our patient’s peritoneovenous shunt accomplished remarkable clinical improvement.

Following resection for cholangiocarcinoma, adjuvant chemotherapy is usually considered.^29)^ Our patient seemed physically able to tolerate adjuvant chemotherapy after shunt creation. However, 4 months had passed since hepatectomy, and adjuvant chemotherapy should be started within 3 months following surgical resection. In the ASCOT trial (JCOG1202),^29)^ adjuvant chemotherapy required initiation within 10 weeks after surgery. We also considered that recurrence of chylous ascites could result from additional cancer treatment, including chemotherapy. Accordingly, adjuvant chemotherapy was not performed in this case.

Treatment for 5 reported patients, including ours, who developed chylous ascites after liver resection is summarized in **Table 2**.^7,30–32)^ Among these, 2 had undergone lymphadenectomy. No site of leakage was established before treatment, and in only 1 was the site identified during surgical repair. Two of the 5 patients were successfully treated with conservative therapy, 1 underwent lymphangiography, and 1 underwent surgery twice. Peritoneovenous shunting was performed only in our case. This procedure has been associated with a high rate of serious complications such as hypokalemia, sepsis, small bowel obstruction, disseminated intravascular coagulation, and air embolism. Additionally, the highly viscous nature of chyle carries a risk of shunt occlusion.^33)^ Our patient developed signs of infection shortly after discharge from the hospital, but the post-treatment course was otherwise uneventful. Peritoneovenous shunting can resolve ascites permanently, often allowing shunt removal.^34)^ However, long-term patency of a Denver shunt has been reported in approximately 30% of cases, while the duration is 2 months in another report.^35)^ In addition, 45% of Denver shunts have been reported to occlude within 1 month in cases of ascites induced by malignant disease.^36)^ Accordingly, the role of such a shunt may be temporary control of ascites to improve a patient’s general condition and nutritional status. Additional definitive treatment may be performed after clinical improvement, especially in patients with a relatively favorable long-term prognosis. While hepatic lymphorrhea associated with injury to the hepatoduodenal ligament is extremely rare^37–39)^ and chylous ascites typically is painless, progressive abdominal distention can persist for weeks to months. Patients may also complain of symptoms such as weight gain, and shortness of breath may occur because of increased abdominal pressure. In all instances, treatment should be tailored to the patient’s condition.

**Table 2:** Summary of reported cases

Author	Age	Sex	Primary disease	Surgical procedure	POD	Detailed examination	Location of leakage	Treatment
Prabhakaran et al.^7)^ (2004)	1	Male	Hepatoblastoma	Rt. hepatectomy	5	None	Not identified	Dietary measures, TPN along with the use of octreotide
Yamamoto et al.^30)^ (2018)	74	Female	Cholangiocarcinoma	Rt. hepatectomy	42	Lymphangiography	Not identified	Lymphangiography
Gallegos et al.^31)^ (2024)	49	Female	CRLM	Ext. rt. hepatectomy (ALPPS)	7	None	Not identified	Dietary measures, TPN along with the use of octreotide
Morino et al.^32)^ (2024)	73	Female	Cholangiocarcinoma	Ext. rt. hepatectomy + lymphadenectomy	50	Lymphoscintigraphy	Not identified	Surgical repair
Our case	80	Male	Cholangiocarcinoma	Ext. rt. hepatectomy + lymphadenectomy	19	Lymphangiography	Not identified	Peritoneovenous shunt

ALPPS, associating liver partition and portal vein ligation for stage hepatectomy; CRLM, colorectal liver metastasis; ext, extended; rt., right; POD, postoperative day; TPN, total parenteral nutrition

## CONCLUSIONS

When chylous ascites is difficult to control after liver resection, treatment modalities such as peritoneovenous shunting can improve control.

## References

[1] Press OW, Press NO, Kaufman SD. Evaluation and management of chylous ascites. Ann Intern Med 1982; 96: 358–64. 7059101 10.7326/0003-4819-96-3-358

[2] Browse NL, Wilson NM, Russo F, et al. Aetiology and treatment of chylous ascites. Br J Surg 1992; 79: 1145–50. 1467885 10.1002/bjs.1800791110

[3] Tanaka K, Ohmori Y, Mohri Y, et al. Successful treatment of refractory hepatic lymphorrhea after gastrectomy for early gastric cancer, using surgical ligation and subsequent OK-432 (Picibanil) sclerotherapy. Gastric Cancer 2004; 7: 117–21. 15224199 10.1007/s10120-004-0276-5

[4] Allen W, Parrott TS, Saripkin L, et al. Chylous ascites following retroperitoneal lymphadenectomy for granulosa cell tumor of the testis. J Urol 1986; 135: 797–8. 3959207 10.1016/s0022-5347(17)45858-7

[5] Leport J, Devars Du Mayne JF, Hay JM, et al. Chylous ascites and encapsulating peritonitis: unusual complications of spontaneous bacterial peritonitis. Am J Gastroenterol 1987; 82: 463–6. 3578227

[6] Aalami OO, Allen DB, Organ CH Jr. Chylous ascites: a collective review. Surgery 2000; 128: 761–78. 11056439 10.1067/msy.2000.109502

[7] Prabhakaran K, Vidyadhar M, Jahoorahmad P, et al. Chylous ascites following liver resection—case report. Pediatr Surg Int 2004; 20: 719–21. 15517286 10.1007/s00383-004-1281-9

[8] Chen J, Lin RK, Hassanein T. Use of orlistat (xenical) to treat chylous ascites. J Clin Gastroenterol 2005; 39: 831–3. 16145348 10.1097/01.mcg.0000177232.51888.2e

[9] Berzigotti A, Magalotti D, Cocci C, et al. Octreotide in the outpatient therapy of cirrhotic chylous ascites: a case report. Dig Liver Dis 2006; 38: 138–42. 16389001 10.1016/j.dld.2005.05.013

[10] Ota H, Miyazawa T, Inaba H, et al. A case report of intractable ascites due to hepatic lymphorrhea from hepatoduodenal ligament after radical gastrectomy for gastric cancer. Jpn J Gastroenterol Surg 1993; 26: 1115–9. (in Japanese with English abstract)

[11] Tomiyasu S, Hirota M, Ohsima H, et al. Estimation of ICG-R_15_ from the parameters of ^99m^Tc-GSA liver scintigraphy and application for hepatectomy. Jpn J Gastroenterol Surg 2000; 33: 579–83.

[12] Yamanaka N, Okamoto E, Oriyama T, et al. A prediction scoring system to select the surgical treatment of liver cancer. Further refinement based on 10 years of use. Ann Surg 1994; 219: 342–6. 8161258 10.1097/00000658-199404000-00003 PMC1243149

[13] Nagino M, Kamiya J, Nishio H, et al. Two hundred forty consecutive portal vein embolizations before extended hepatectomy for biliary cancer: surgical outcome and long-term follow-up. Ann Surg 2006; 243: 364–72. 16495702 10.1097/01.sla.0000201482.11876.14 PMC1448943

[14] Liver Cancer Study Group of Japan. General rules for the clinical and pathological study of primary liver cancer, 3rd ed. Tokyo: Kanehara & Co., 2010.

[15] Inoue M, Nakatsuka S, Yashiro H, et al. Lymphatic intervention for various types of lymphorrhea: access and treatment. Radiographics 2016; 36: 2199–211. 27831840 10.1148/rg.2016160053

[16] Patil AR, Nandikoor S, De Marco J, et al. Disorders of the lymphatic system of the abdomen. Clin Radiol 2016; 71: 941–52. 27450410 10.1016/j.crad.2016.06.116

[17] Davis C. Textbook of surgery. Philadelphia: Saunders; 1978. p.1794–8.

[18] Bhardwaj R, Vaziri H, Gautam A, et al. Chylous ascites: a review of pathogenesis, diagnosis and treatment. J Clin Transl Hepatol 2018; 6: 105–13. 29577037 10.14218/JCTH.2017.00035 PMC5863006

[19] Kojima M, Inoue M, Yamamoto S, et al. Successful treatment of hepatic lymphorrhea by percutaneous transhepatic lymphangiography followed by sclerotherapy using OK-432. Surg Case Rep 2019; 5: 203. 31872305 10.1186/s40792-019-0761-z PMC6928181

[20] Cárdenas A, Chopra S. Chylous ascites. Am J Gastroenterol 2002; 97: 1896–900. 12190151 10.1111/j.1572-0241.2002.05911.x

[21] Almakdisi T, Massoud S, Makdisi G. Lymphomas and chylous ascites: review of the literature. Oncologist 2005; 10: 632–5. 16177287 10.1634/theoncologist.10-8-632

[22] Lee EW, Shin JH, Ko HK, et al. Lymphangiography to treat postoperative lymphatic leakage: a technical review. Korean J Radiol 2014; 15: 724–32. 25469083 10.3348/kjr.2014.15.6.724 PMC4248627

[23] Kariya S, Komemushi A, Nakatani M, et al. Intranodal lymphangiogram: technical aspects and findings. Cardiovasc Intervent Radiol 2014; 37: 1606–10. 24722896 10.1007/s00270-014-0888-z

[24] Shamohammadi M, Ramezani A, Naseh E, et al. Management of chylous ascites following pancreaticoduodenectomy surgery using radiotherapy: a case report and review of literature. Int J Surg Case Rep 2025; 134: 111640. 40694921 10.1016/j.ijscr.2025.111640 PMC12301827

[25] Kawasaki R, Sugimoto K, Fujii M, et al. Therapeutic effectiveness of diagnostic lymphangiography for refractory postoperative chylothorax and chylous ascites: correlation with radiologic findings and preceding medical treatment. AJR Am J Roentgenol 2013; 201: 659–66. 23971461 10.2214/AJR.12.10008

[26] Onodera T, Goseki N, Kosaki G. Prognostic nutritional index in gastrointestinal surgery of malnourished cancer patients. Nihon Geka Gakkai Zasshi 1984; 85: 1001–5. (In Japanese with English abstract). 6438478

[27] Tsauo J, Shin JH, Han K, et al. Transjugular intrahepatic portosystemic shunt for the treatment of chylothorax and chylous ascites in cirrhosis: a case report and systematic review of the literature. J Vasc Interv Radiol 2016; 27: 112–6. 26723922 10.1016/j.jvir.2015.09.022

[28] Makino Y, Shimanuki Y, Fujiwara N, et al. Peritoneovenous shunting for intractable chylous ascites complicated with lymphangioleiomyomatosis. Intern Med 2008; 47: 281–5. 18277030 10.2169/internalmedicine.47.0475

[29] Nakachi K, Ikeda M, Konishi M, et al. Adjuvant S-1 compared with observation in resected biliary tract cancer (JCOG1202, ASCOT): a multicentre, open-label, randomised, controlled, phase 3 trial. Lancet 2023; 401: 195–203. 36681415 10.1016/S0140-6736(22)02038-4

[30] Yamamoto R, Mokuno Y, Matsubara H, et al. Chylothorax after hepatectomy: a case report. J Med Case Rep 2018; 12: 347. 30474568 10.1186/s13256-018-1882-x PMC6260677

[31] Gallegos LEG, Velázquez CAC, Azuela OC, et al. Chylous ascites after associating liver partition and portal vein ligation for stage hepatectomy (ALPPS): overview and case report. J Surg Case Rep 2024; 2024: rjae357. 38817794 10.1093/jscr/rjae357 PMC11138119

[32] Morino K, Morimura Y, Tanaka H, et al. Refractory chylous ascites leading to chylothorax following extended right hepatectomy for intrahepatic cholangiocarcinoma. Cureus 2024; 16: e73301. 39655122 10.7759/cureus.73301 PMC11625965

[33] Al-Busafi SA, Ghali P, Deschênes M, et al. Chylous ascites: evaluation and management. ISRN Hepatol 2014; 2014: 240473. 27335837 10.1155/2014/240473 PMC4890871

[34] Yarmohammadi H, Brody LA, Erinjeri JP, et al. Therapeutic application of percutaneous peritoneovenous (Denver) shunt in treating chylous ascites in cancer patients. J Vasc Interv Radiol 2016; 27: 665–73. 26965362 10.1016/j.jvir.2015.12.014 PMC5060070

[35] Huang Y, Gloviczki P, Duncan AA, et al. Managment of refractory chylous ascites with peritoneovenous shunts. J Vasc Surg Venous Lymphat Disord 2017; 5: 538–46. 28623993 10.1016/j.jvsv.2017.03.011

[36] Sugawara S, Sone M, Arai Y, et al. Radiological insertion of Denver peritoneovenous shunts for malignant refractory ascites: a retrospective multicenter study (JIVROSG-0809). Cardiovasc Intervent Radiol 2011; 34: 980–8. 21191592 10.1007/s00270-010-0057-y

[37] Matsumoto S, Mori H, Tada I. Successful demonstration of post-operative lymphatic fistula by percutaneous transhepatic lymphography. Clin Radiol 2000; 55: 485–6. 10873699 10.1053/crad.2000.0123

[38] Guez D, Nadolski GJ, Pukenas BA, et al. Transhepatic lymphatic embolization of intractable hepatic lymphorrhea. J Vasc Interv Radiol 2014; 25: 149–50. 24365510 10.1016/j.jvir.2013.09.002

[39] Inaba Y, Arai Y, Matsueda K, et al. Intractable massive ascites following radical gastrectomy, treatment with local intraperitoneal administration of OK-432 using a unified CT and fluoroscopy system. Australas Radiol 2003; 47: 465–7. 14641206 10.1046/j.1440-1673.2003.01223.x

